# In vivo evaluation of a novel ^18^F-labeled PET radioligand for translocator protein 18 kDa (TSPO) in monkey brain

**DOI:** 10.1007/s00259-023-06270-9

**Published:** 2023-05-30

**Authors:** Xuefeng Yan, Fabrice G. Siméon, Jeih-San Liow, Cheryl L. Morse, Jose A. Montero Santamaria, Madeline Jenkins, Lester S. Manly, Maia Van Buskirk, Sami S. Zoghbi, Victor W. Pike, Robert B. Innis, Paolo Zanotti-Fregonara

**Affiliations:** Molecular Imaging Branch, National Institute of Mental Health, NIH, 10 Center Drive, Bethesda, MD 20892 USA

**Keywords:** [^18^F]SF51, TSPO, PET, Brain, Monkey

## Abstract

**Purpose:**

[^18^F]SF51 was previously found to have high binding affinity and selectivity for 18 kDa translocator protein (TSPO) in mouse brain. This study sought to assess the ability of [^18^F]SF51 to quantify TSPO in rhesus monkey brain.

**Methods:**

Positron emission tomography (PET) imaging was performed in monkey brain (n = 3) at baseline and after pre-blockade with the TSPO ligands PK11195 and PBR28. TSPO binding was calculated as total distribution volume corrected for free parent fraction in plasma (*V*_T_/*f*_P_) using a two-tissue compartment model. Receptor occupancy and nondisplaceable uptake were determined via Lassen plot. Binding potential (*BP*_ND_) was calculated as the ratio of specific binding to nondisplaceable uptake. Time stability of *V*_T_ was used as an indirect probe to detect radiometabolite accumulation in the brain. In vivo and ex vivo experiments were performed in mice to determine the distribution of the radioligand.

**Results:**

After [^18^F]SF51 injection, the concentration of brain radioactivity peaked at 2.0 standardized uptake value (SUV) at ~ 10 min and declined to 30% of the peak at 180 min. *V*_T_/*f*_P_ at baseline was generally high (203 ± 15 mL· cm^−3^) and decreased by ~ 90% after blockade with PK11195. *BP*_ND_ of the whole brain was 7.6 ± 4.3. *V*_T_ values reached levels similar to terminal 180-min values by 100 min and remained relatively stable thereafter with excellent identifiability (standard errors < 5%), suggesting that no significant radiometabolites accumulated in the brain. Ex vivo experiments in mouse brain showed that 96% of radioactivity was parent. No significant uptake was observed in the skull, suggesting a lack of defluorination in vivo.

**Conclusion:**

The results demonstrate that [^18^F]SF51 is an excellent radioligand that can quantify TSPO with a good ratio of specific to nondisplaceable uptake and has minimal radiometabolite accumulation in brain. Collectively, the results suggest that [^18^F]SF51 warrants further evaluation in humans.

**Supplementary information:**

The online version contains supplementary material available at 10.1007/s00259-023-06270-9.

## Introduction

The 18 kDa translocator protein (TSPO) is an inflammation-related protein that is widely expressed not only in microglia, but also in neurons, astrocytes, and blood vessels. The physiological role of this protein remains enigmatic, especially considering recent data that suggest that, contrarily to rodents, humans do not overexpress TSPO transcripts and proteins in microglia of cases with Alzheimer's disease, amyotrophic lateral sclerosis, and multiple sclerosis relative to controls [1]. Nevertheless, TSPO remains the most studied biomarker of neuroinflammation using positron emission tomography (PET) in neurologic [2] and psychiatric [3] disorders. The first PET radioligand for TSPO was [^11^C]PK11195 [4], which suffered from low specific signal and high nonspecific binding [5]. Although second-generation radioligands such as [^11^C]PBR28 [6] and [^11^C]DPA713 [7] had much higher specific binding than [^11^C]PK11195, all of these radioligands, including [^11^C]PK11195 [8], were sensitive to a co-dominantly expressed polymorphism of the three identified genotypes—high affinity binders (HABs), low affinity binders (LABs), and mixed affinity binders (MABs). LABs, which comprise 5–10% in the US and European populations, were found to have immeasurably low specific binding and thus needed to be excluded a priori from PET scans [5].

In the search for improved TSPO radioligands less sensitive to genotype, our laboratory developed the third-generation TSPO radioligand [^11^C]ER176 [9]. However, [^11^C]ER176 only partially overcame this problem. Although it has relatively lower sensitivity to genotype than [^11^C]PBR28, the sensitivity to genotype was high enough that it had to be corrected a posteriori. The unexpected advantage of [^11^C]ER176 was that it provided accurate values for LABs [10] due to a lack of brain uptake of radiometabolites. As background, the accumulation of radiometabolites in brain can be indirectly measured as increasing values over time of the apparent receptor density (distribution volume (*V*_T_)). Unlike [^11^C]PBR28 and [^11^C]DPA713, [^11^C]ER176 provided *V*_T_ values that were stable over increasing lengths of scanning [5]. Thus, while [^11^C]ER176 was sensitive to genotype, it was nevertheless an advance over the second-generation radioligands [^11^C]PBR28 and [^11^C]DPA713 because it provided accurate, time-stable *V*_T_ values in all three genotypes. Most notably, LABs did not need to be excluded prior to PET scanning using [^11^C]ER176; instead, the effect of genotype was corrected after the PET scan.

Building on this work, this study sought to develop an ^18^F-labeled analog of [^11^C]ER176 [11] because the longer half-life of ^18^F (109.8 min) compared to ^11^C (20.4 min) would allow it to be produced at a central radiopharmacy and distributed to distant imaging sites. Towards that end, six analogs of ER176 were synthesized that could be labeled with either ^11^C or ^18^F: three isomers with a fluoro group and three with a trifluoromethyl group at one of three positions (*ortho*, *meta*, and *para*) of the pendant aryl ring (Supplementary Fig. S1). Because radiolabeling these analogs was much easier using ^11^C than ^18^F, the performance of the six ^11^C-labeled analogs were compared in order to quantify TSPO in monkey brain [12]. The ^11^C labeled analog with ^18^F-fluorine in the *meta* position of the pendant aryl ring ([^11^C]SF12051, hereafter abbreviated to [^11^C]SF51) had a high ratio of specific to background uptake (whole brain *BP*_ND_ = 8.1), excellent quantitation by compartmental modeling, and seemed suitable to extend to human participants.

However, while [^11^C]SF51 performed well in nonhuman primates [12], it was not clear that [^18^F]SF51 would do so because of possibly differential disposition of the radiolabeled metabolites. ^11^C and ^18^F labels are located at different positions of the molecule, and metabolism may generate different radiometabolites; some of these radiometabolites might accumulate in brain or its adjacent structures. For example, [^18^F]SF51 defluorination could generate ^18^F-fluoride ions that could accumulate in the skull and that might thus contaminate quantitation of adjacent brain tissue. In contrast, [^11^C]SF51 defluorination would generate nonradioactive fluoride ions. Thus, before proceeding to a first-in-human study using [^18^F]SF51, it was necessary to ensure that this radioligand performs well in monkeys, which provide the best animal model for humans.

This study sought to assess the ability of [^18^F]SF51 to quantify TSPO in rhesus monkey brain. The density of the target (*V*_T_) was measured with compartmental modeling of the concentration of radioactivity in brain relative to the concentration of parent radioligand, separated from radiometabolites, in arterial plasma. Scans were performed at baseline and after pharmacological blockade to assess the specific binding of the radioligand.

## Material and methods

### Radiochemistry

Synthesis of [^18^F]SF51 from a diaryliodonium salt [11] has previously been described. For this study, [^18^F]SF51 was prepared by a new method based on radiofluorination of a diarylselenone precursor according to a protocol recorded in FDA-sanctioned eIND #162,310. Detailed information regarding synthesis of the precursor and radiosynthesis will be published separately.

### Animals

All studies were conducted in accordance with the ARRIVE guidelines for reporting animal research as well as the *Guidelines for the Care and Use of Laboratory Animals, 8th Edition* [13, 14]. All studies were approved by the National Institute of Mental Health Animal Care and Use Committee.

### PET study in rhesus monkeys

Three male rhesus monkeys (10.0 ± 1.8 kg) were initially immobilized with ketamine hydrochloride (1 mg/kg, i.m.), anesthesia was maintained with 1.0–3.0% isoflurane and 98% O_2_, and body temperature was maintained at between 37.0–37.5 °C. Electrocardiogram, body temperature, heart rate, and respiratory rate measures were monitored throughout the scan.

Brain PET imaging was performed using a microPET Focus 220 camera (Siemens Medical Solution; Knoxville, TN). Following a transmission scan using a ^57^Co point source, 180-min dynamic PET scans were acquired after IV injection of [^18^F]SF51 (198 ± 32 MBq, Supplementary Table S1). For baseline experiments, 20 mL of a vehicle containing 20% (2-hydroxypropyl)-β-cyclodextrin and two equivalents of HCl were injected intravenously 10 min before radioligand administration. For the blocked experiment, PK11195 was dissolved in vehicle and then injected intravenously (5 mg/kg) 10 min before radioligand injection for all three monkeys [15, 16]. One of the three monkeys underwent an additional blocked scan with 5 mg/kg of PBR28, performed using a MultiScan™ LFER 150 PET/CT (Mediso USA, LLC; Arlington, VA). The interval between baseline and blocked imaging sessions was at least three weeks in order to allow the monkey to recover from arterial blood sampling. Focus 220 PET images were histogrammed into 30 time frames (6 × 30, 3 × 60, 2 × 120, 4 × 300, and 15 × 600 s) and reconstructed using Fourier rebinning plus a two-dimensional filtered back-projection with scatter and attenuation correction. MultiScan LFER PET images were reconstructed with 3D-OSEM with two iterations and nine subsets and were corrected for scatter and attenuation.

### Parent radioactivity and radiometabolite analysis in plasma

Arterial blood sampling was performed in all scans to determine plasma parent concentration of the radioligand. Seventeen blood samples were drawn from an implanted port in the femoral artery of the monkeys during the 180-min PET scans at 15-s intervals for the first two minutes, followed by samples at 3, 5, 10, 30, 60, 90, 120, 150, and 180 min (volumes varied from 1.0 to 5.0 mL). Radioactivity in plasma was quantified and corrected for radiometabolites using high-performance liquid chromatography (HPLC). The separation was performed on an X-Terra *C*_18_ column (10 µm, 7.8 × 300 mm) with an isocratic mobile phase composed of methanol: water: triethylamine (77.5:22.5:0.1 by vol.) at a flow rate of 5 mL/min. The plasma free fraction (*f*_P_) was measured by ultrafiltration, as previously described [17, 18]. A standard *f*_P_ was measured in a frozen aliquot of pooled plasma in parallel with the experimental blood sample, and as internal standard it was then used to normalize the measured *f*_P_ of the blood samples.

### Kinetic analysis

PET images were co-registered to a standardized monkey MRI template using PMOD software (PMOD version 4.1, PMOD Technologies Ltd.; Zurich, Switzerland). Thirty-three predefined regions of interest (ROIs) from a monkey brain template were applied to the co-registered PET images to obtain regional time-activity curves (Supplementary Table S2). Brain uptake was expressed as standardized uptake value (SUV), which normalizes for injected radioactivity and body weight. Using the brain time-activity curves and the radiometabolite-corrected arterial input function, the total *V*_T_ was derived from a two-tissue compartment model (2TCM). Receptor occupancy by PK11195 and nondisplaceable distribution volume (*V*_ND_/*f*_P_) of [^18^F]SF51 were determined by an axes-transformed Lassen plot [19, 20], where the y-axis reports the difference in *V*_T_ between the baseline and the blocked scans, corrected by the respective *f*_p_ values, and the x-axis reports the values at baseline (*V*_Tbase_/*f*_Pbase_). Although the standard representation of the axes-transformed Lassen plot does not include correction for *f*_P_, the mathematical justification for this approach is given as follows:

The equation of the axes-transformed Lassen plot, corrected by *f*_P_, is:

1 $${V}_{\mathrm{Tbase}}/{f}_{\mathrm{Pbase}}- {V}_{\mathrm{Tblock}}/{f}_{\mathrm{Pblock}} =\mathrm{ Slope }\bullet ({V}_{\mathrm{Tbase}}/{f}_{\mathrm{Pbase}} -\mathrm{ x}-\mathrm{intercept})$$

In the baseline and blocked scans, the following equations can be derived:

2 $${V}_{\mathrm{Tbase}}/{f}_{\mathrm{Pbase}} = {V}_{\mathrm{S}}/{f}_{\mathrm{Pbase}} + {V}_{\mathrm{ND}}/{f}_{\mathrm{Pbase}}$$

3 $${V}_{\mathrm{Tblock}}/{f}_{\mathrm{Pblock}} = {V}_{\mathrm{S}}/{f}_{\mathrm{Pbase}} (1-\mathrm{ Occupancy}) + {V}_{\mathrm{ND}}/{f}_{\mathrm{Pbase}}$$

Based on Eqs. 2 and 3, the following equation can be derived:

4 $${V}_{\mathrm{Tbase}}/{f}_{\mathrm{Pbase}} - {V}_{\mathrm{Tblock}}/{f}_{\mathrm{Pblock}} =\mathrm{ Occupancy }\cdot ({V}_{\mathrm{Tbase}}/{f}_{\mathrm{Pbase}} - {V}_{\mathrm{ND}}/{f}_{\mathrm{Pbase}})$$

From Eqs. 1 and 4, the following conclusions can be drawn:

$$\mathrm{Slope }=\mathrm{ Occupancy}$$

$$\mathrm{x}-\mathrm{intercept }= {V}_{\mathrm{ND}}/{f}_{\mathrm{Pbase}}$$

*V*_Tbase_/*f*_Pbase_ stands for the total distribution volume in the baseline state. This is the condition before the administration of the competing drug, where all radioligand binding sites are available; *V*_Tblock_/*f*_Pblock_, represents the total distribution volume in the blocked state. This is after the competing drug is introduced, and some of the binding sites are occupied by the drug, reducing the overall distribution volume; *V*_S_/*f*_Pbase_ is the *f*_P_ corrected specific volume of distribution; *V*_ND_/*f*_Pbase_ is the *f*_P_ corrected non-displaceable volume of distribution; Occupancy represents the fraction of radioligand binding sites occupied by the competing drug.

To determine the minimum scan duration needed to reliably measure *V*_T_ as well as to indirectly assess whether radiometabolites accumulate in the brain, the time stability of *V*_T_ was examined by truncating the scan duration from 180 min down to 20 min in 20-min increments.

### PET study in mice

Imaging experiments were performed on a microPET Focus 120 camera (Siemens Medical Solution; Knoxville, TN). Mice were anesthetized with 1.5% isoflurane in oxygen at a flow rate of 1 L/min. [^18^F]SF51 (150 µL; 2.9 ± 0.6 MBq) was injected intravenously through the tail vein of each mouse. Baseline scans were obtained in 12 mice, and another eight mice were imaged after pre-administration of 5 mg/kg PK11195. Scans were acquired for 120 min. Data were histogrammed into 24 time frames (6 × 20, 5 × 60, 4 × 120, 3 × 300, 3 × 600 and 3 × 1200 s) and reconstructed using Fourier rebinning plus a two-dimensional filtered back projection. No scatter or attenuation corrections were applied. PET images were analyzed using PMOD with a single ROI of the whole brain.

An ex vivo experiment was performed in another mouse 120 min after [^18^F]SF51 injection. Anticoagulated blood was withdrawn from the myocardium, and the brain was extracted. The harvested brain was weighed and immediately underwent radioanalysis. The plasma was separated by centrifugation, and the radioactivity in both whole blood and plasma was measured as previously described [17].

### Statistical analysis

All statistical analyses were performed in GraphPad Prism (version 5.02 GraphPad Software, Inc, San Diego, California). Results with p-values < 0.05 were considered statistically significant. Data are presented as mean ± standard deviation (SD) or mean with a range.

## Results

### Radiochemistry

**Fig. 1:**
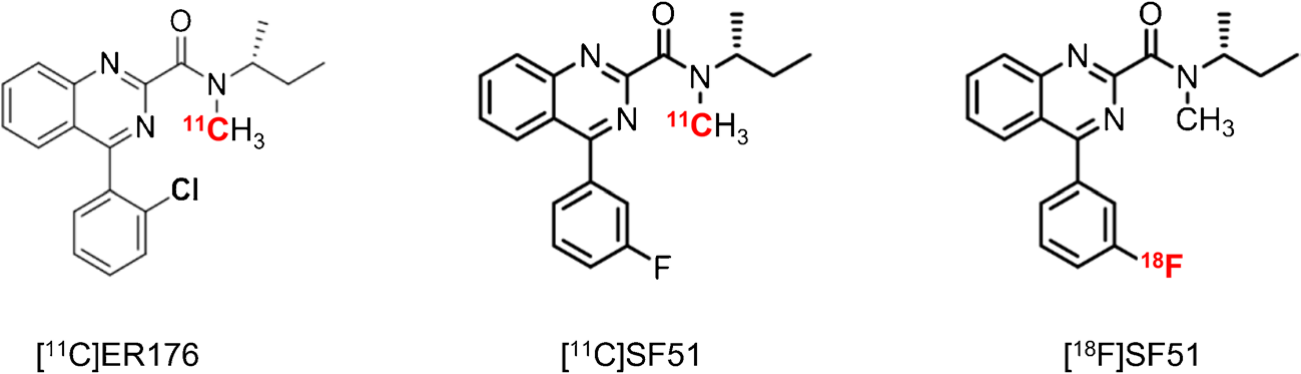
Chemical structures of [^11^C]ER176, [^11^C]SF51, and [.^18^F]SF51

Radiochemically stable [^18^F]SF51 was obtained for intravenous injection in sterile saline containing ethanol (10% v/v) in a total volume of 10 mL (Fig. 1). All preparations of [^18^F]SF51 had high radiochemical purity (> 99% by radio HPLC) and molar activity between 88.5 and 126 GBq/mmol (2.39 – 3.42 Ci/mmol).

### Arterial input function

**Fig. 2:**
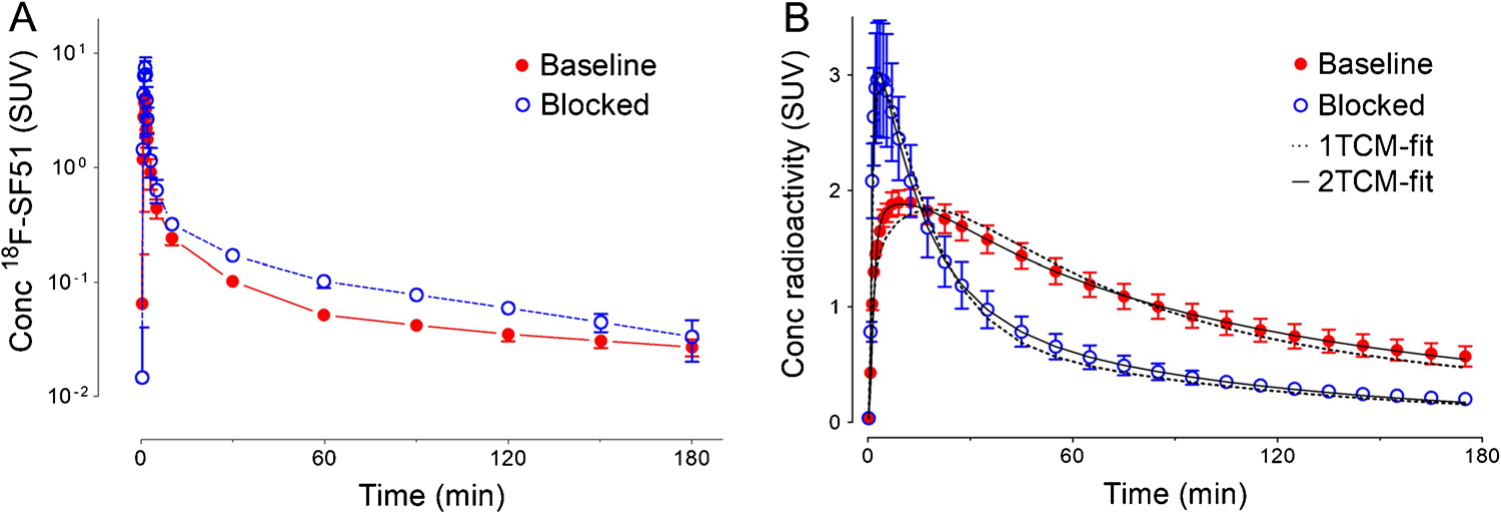
Concentration of [^18^F]SF51 in arterial plasma (**A**) and of radioactivity in whole brain (**B**) of rhesus macaques at baseline and after pre-blockade with PK11195. The selective 18 kDa translocator protein (TSPO) ligand PK11195 (5 mg/kg i.v.) was injected 10 min prior to [^18^F]SF51. Concentration was expressed as standardized uptake value (*SUV*). Symbols and error bars represent mean and SD (n = 3), respectively. The brain time-activity curves were fitted well by visual inspection using a 2TCM, but not by1TCM

The [^18^F]SF51 parent concentration peaked at 4.1 ± 0.4 SUV with a steady washout that was well-fitted by a three-exponential function (Fig. 2A). The blocked plasma concentrations were higher than baseline both at the peak (7.5 ± 1.7 SUV) and during the rest of the curve (Fig. 2A), which was due to the displacement from blood cells and peripheral organs induced by PK11195 administration [8]. The mean *f*_P_ value of blocked scans (18.8 ± 3.6%, n = 3) was almost three-fold higher than that of baseline scans (6.4 ± 0.7%, n = 3).

With HPLC, the average percentage of parent in plasma 30 min post-injection was found to equal that of the radiometabolites. It decreased to 25% at 120 min and then stayed at roughly the same percentage until 180 min post-injection (Supplementary Fig. S2A). At least five radiometabolites were more hydrophilic than the parent in plasma (Supplementary Fig. S2B).

### Brain distribution and kinetics in monkeys

**Fig. 3:**
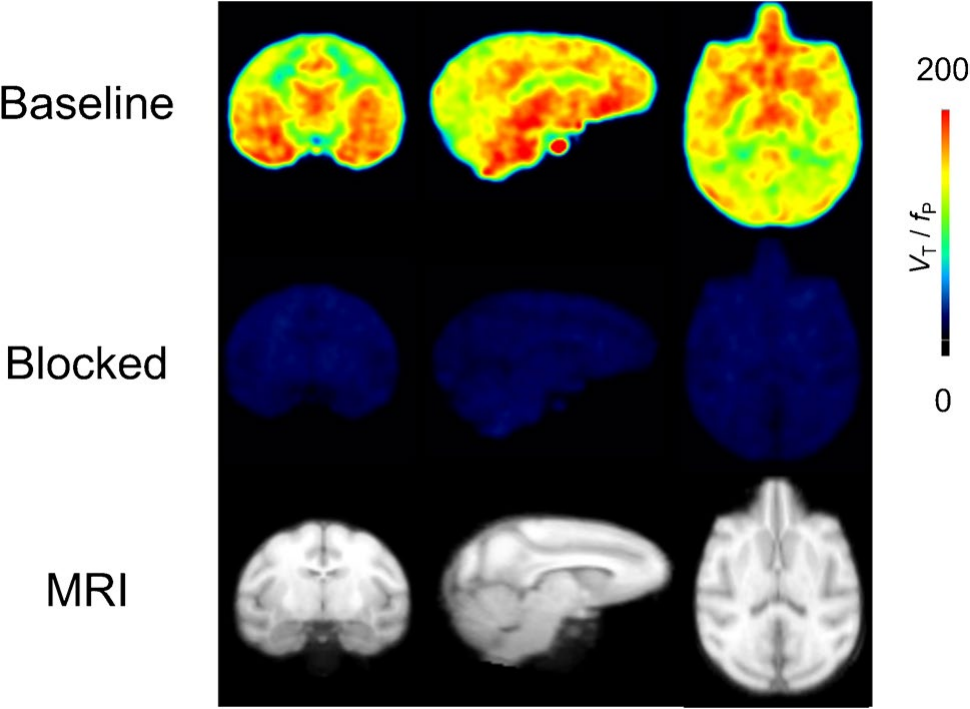
Parametric images of total 18 kDa translocator protein (TSPO) binding (*V*_T_/*f*_P_) for [^18^F]SF51 in monkey brain at baseline (top row) and after PK11195 pre-blockade (5 mg/kg, middle row). The template MRI of monkey brain is shown in the bottom row. Each total distribution volume corrected for free parent fraction in plasma (*V*_T_/*f*_P_) image was generated by Logan Plot using 0–180 min of PET data graphical analysis

At baseline, [^18^F]SF51 readily entered monkey brain; its uptake peaked at five to 15 min post-injection (mean SUV peak 2.0 ± 0.1) and was followed by moderate wash-out (Fig. 2B). Brain activity decreased to 50% of the peak by 110 min and to 30% of the peak by 180 min. The mean SUV peak after pre-blockade with 5 mg/kg PK11195 was higher (3.0 ± 0.5), and the wash-out phase was faster, likely because of reduced retention of the radioligand (Fig. 2B). As expected from the known distribution of TSPO in human and monkey brain [6], the activity was widespread and fairly uniform in the cortical gray matter, cerebellum, and thalamus (Fig. 3). At baseline, a low uptake in the skull was observed (Supplementary Fig. S3) that did not generate significant spill-over at visual analysis. This uptake was blocked by both PK11195 and PBR28 and is therefore likely due to extracerebral specific binding, not to deposition of ^18^F-fluoride ion into bone after defluorination.

### Quantification of [^18^F]SF51 binding in monkey brain

The brain time-activity curves were fitted well by visual inspection using a 2TCM for all the studied regions. However, 1TCM did not adequately fit the time-activity curves (Fig. 2B, Supplementary Fig. S4). Logan-plot gave results consistent with 2TCM, with a negative bias of about 4% and 2% for baseline and blocked scans, respectively (Supplementary Table S3). The regions with the highest uptake were the amygdala, striatum, and insula (*V*_T_/*f*_P_: 242, 225, and 224 mL· cm^−3^, respectively). Cerebellum had the lowest uptake (179 mL· cm^−3^). The coefficients of variation of *V*_T_/*f*_P_, calculated in 11 regions for each monkey, were within 30% (Table 1). *V*_T_/*f*_P_ values were consistent among the three animals, with low inter-individual variability at baseline (10% variability).

**Table 1:** Regional distribution volume corrected for plasma protein binding (*V*_T_/*f*_P_) of [^18^F]SF51 in monkey brain (n = 3)

Region	*V*_T_/*f*_P_ (mL·cm^−3^)					
	Baseline			Blocked		
	Mean	SD	COV	Mean	SD	COV
Whole Brain	203	15	0.1	24	7	0.3
Frontal Cortex	201	11	0.1	26	8	0.3
Cingulate Cortex	218	16	0.1	25	7	0.3
Striatum	225	16	0.1	26	7	0.3
Insula	224	14	0.1	23	8	0.3
Temporal Cortex	209	21	0.1	22	7	0.3
Amygdala	242	22	0.1	23	5	0.2
Hippocampus	220	25	0.1	21	6	0.3
Thalamus	218	20	0.1	27	8	0.3
Parietal Cortex	202	10	0.0	24	7	0.3
Occipital Cortex	183	15	0.1	23	6	0.3
Cerebellum	179	21	0.1	26	8	0.3

*COV* coefficient of variation; *SD* standard deviation

**Fig. 4:**
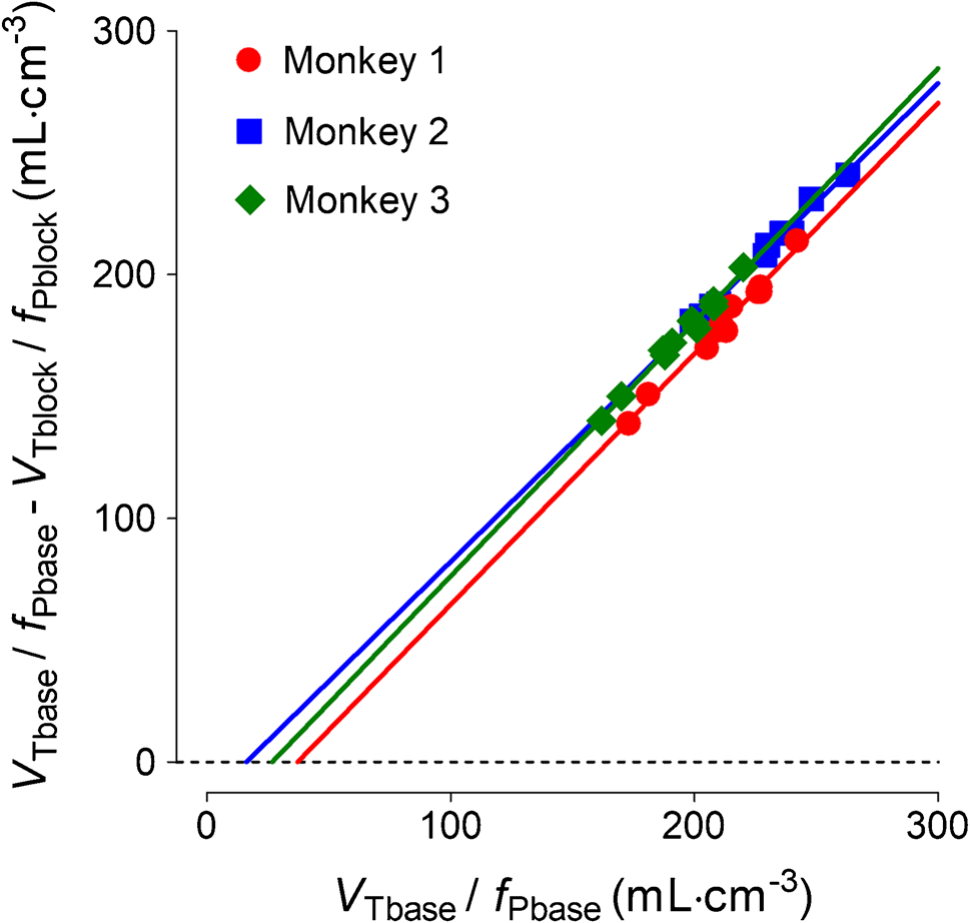
Receptor occupancy and nondisplaceable distribution volume (*V*_ND_/*f*_P_) of [.^18^F]SF51 determined by Lassen plot. Data were from imaging studies in three rhesus monkeys at baseline and after administration of PK11195 at a dose of 5 mg/kg. The slope was 1.02 ± 0.03 and the x-intercept was 27 ± 11 (mean ± SD, n = 3). The R-squared values of linear fit were over 0.98

A global measure of receptor occupancy by PK11195 was calculated using the Lassen plot, and the *V*_T_/*f*_P_ was estimated by 2TCM. The occupancy was 102 ± 3%. *V*_ND_/*f*_P_, determined as the x-intercept of the regression lines, was 27 ± 11 mL· cm^−3^ (Fig. 4). *BP*_ND_ of whole brain was 7.6 ± 4.3, which was comparable to that of [^11^C]SF51 [12]. In all regions, the *K*_1_ values were about 30% higher in blocked scans compared to baseline scans, likely due to changes in blood flow and *f*_P_ (Supplementary Table S4). The brain (and skull) uptake was also blockable after administration of PBR28, whose structure is different from that of either SF51 or PK11195 (Supplementary Table S5).

**Fig. 5:**
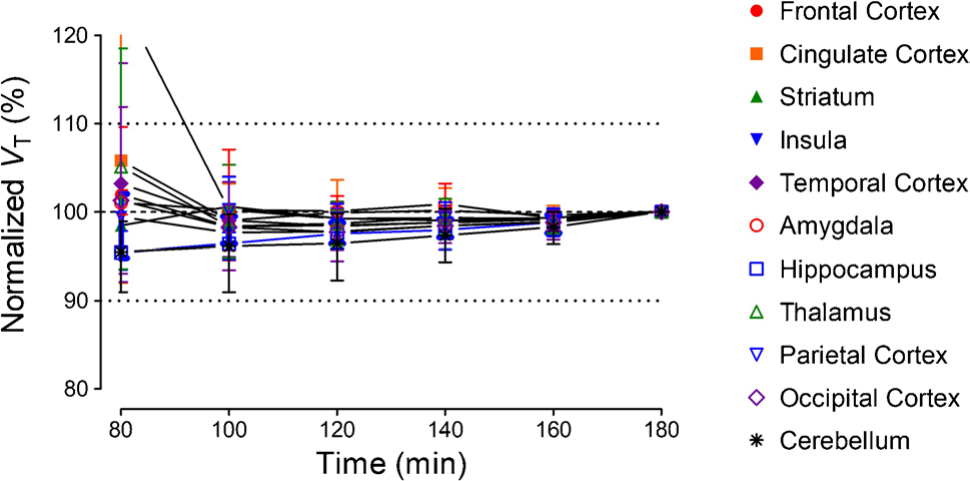
Time-stability analysis of regional total distribution volume (*V*_T_) for [.^18^F]SF51. *V*_T_ was estimated via two-tissue compartment modeling and normalized to the terminal *V*_T_ value at 180 min. Symbols and error bars represent the mean and SD (n = 3)

*V*_T_ values of the various regions were stable within 100 min of imaging. That is, *V*_T_ values were within 10% of that at 180 min, which suggests it requires at least 100 min to reliably measure *V*_T_. It also suggests that radiometabolites were not accumulating in the brain (Fig. 5).

### Mouse study

**Fig. 6:**
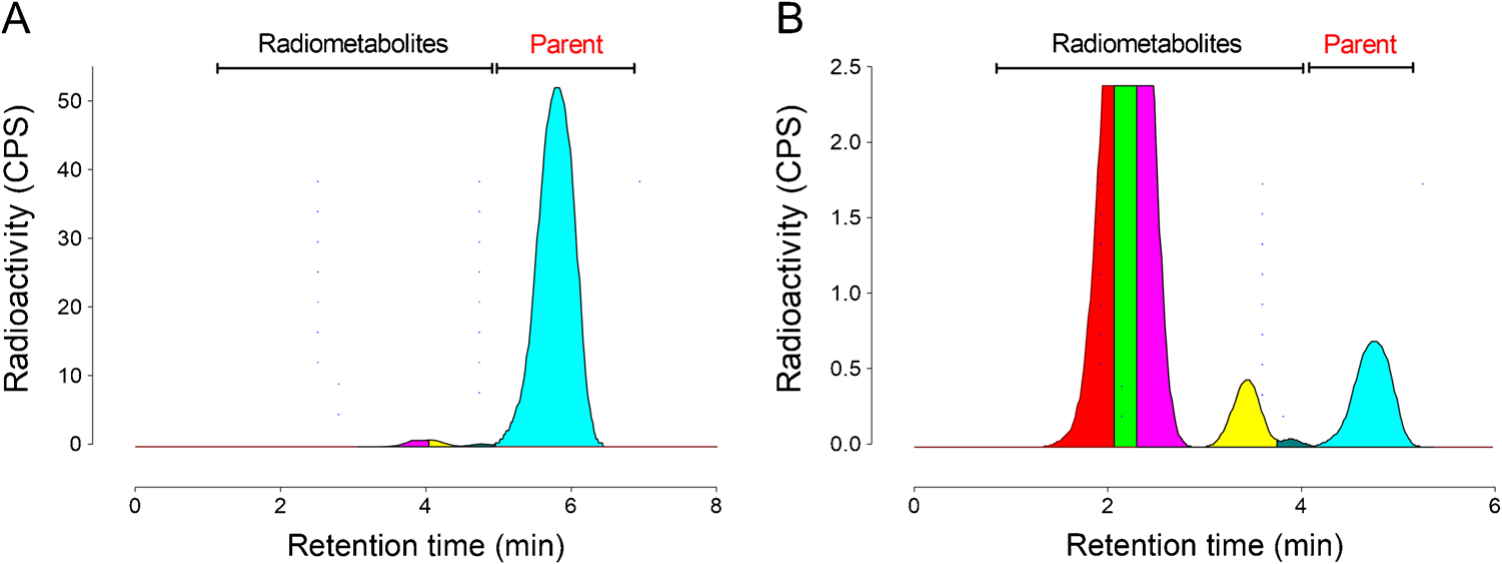
Ex vivo radio-chromatograms in mice 120 min after injection. Parent was 96.4% in brain (**A**) but only 9.1% in plasma (**B**)

As previously described [11], PET images showed about 80% blockade of [^18^F]SF51 in mouse brain after i.v. administration of 5 mg/kg PK11195. Brain uptake at baseline reached peak values of ~ 0.8 SUV at around 15 min and moderately washed out thereafter (Supplementary Fig. S5A). After blockade with PK11195, brain uptake reached a peak of ~ 2.5 SUV within three minutes and then washed out very rapidly. In the last 80 min of scanning, brain activity after blockade was 25% of that at baseline (Supplementary Fig. S5B). The blockable high uptake (> 2 SUV) was also found in lung, which is known to contain high levels of TSPO. No significant increase in radioactivity was detected in bone. Parent radioligand represented 96.4% of radioactivity in the brain but only 9.1% of radioactivity in plasma (Fig. 6), suggesting that radiometabolites generated in the plasma did not significantly enter the brain.

## Discussion

This study demonstrated the ability of [^18^F]SF51 to quantify TSPO density in rhesus monkey brain. The radioligand readily crossed the blood–brain barrier and had visually high uptake in the brain. Indeed, the average *V*_T_/*f*_P_ in the whole brain was 203 mL· cm^−3^, which is in the same range of that of its close analog [^11^C]ER176 (186 mL·cm^−3^) [16] and of [^11^C]PBR28 (287 mL·cm^−3^) [21]. No significant defluorination was observed in the bones of the skull. In addition, distribution of SF51 in brain reflected the known distribution of TSPO and, in particular, reflected the pattern observed using [^11^C]SF51 in our previous primate studies [12]. Regions such as the amygdala, striatum, and cortex displayed high TSPO density, while cerebellum had the lowest binding. Furthermore, pre-blocking with PK11195 displaced about 90% of [^18^F]SF51 binding, which translated into a high *BP*_ND_ (7.6). This *BP*_ND_ value was similar to that observed for [^11^C]SF51 (*BP*_ND_ = 8.1), which shares an identical chemical structure with [^18^F]SF51, as well as that of the structurally similar ER176 (*BP*_ND_ = 8.9) [12]. The specificity of [^18^F]SF51 binding was also corroborated by the mouse study, which showed a clear blockable effect not only in the brain, but also in the lungs, which are known to contain high levels of TSPO.

*V*_T_ in the brain was well-identified using a 2TCM, and its value was relatively stable after 80 min of acquisition. This suggests that, despite position of the radiolabel in [^18^F]SF51 differing from that in [^11^C]SF51, no significant radiometabolite accumulated in the brain. In addition, the ex vivo mouse study showed that the radiometabolites found in plasma entered the brain at only negligible concentrations.

Uptake in the skull was blocked by both PK11195 and PBR28, the latter having a very different chemical structure compared to both SF51 and PK11195. Therefore, this uptake is likely due to specific binding in the extracerebral space and not to deposition of ^18^F-fluoride ion into bone after defluorination or to binding to extra-target biding sites. A similar skull blockade was observed also in the ^11^C version of the compound. Monkey [^11^C]SF51 data were acquired in Lee et al. [16] and reanalyzed for the present work (Supplementary Figure S6).

Plasma free fraction (*f*_P_) is directly related to the ability of the radioligand to cross the blood–brain barrier and bind to its target [22]. Our measurements of *f*_P_ varied between baseline (6 ± 1%) and blocked scans (19 ± 4%), but we are confident that these measurements were accurate because of the consistency of *f*_P_ measurements drawn from an internal standard used in each study. Specifically, *f*_P_ was measured in a frozen aliquot of pooled plasma in parallel with the experimental blood samples, and the *f*_P_ values of this internal standard were then used to correct those of the blood samples. For the six PET scans reported in this paper (two per monkey), the *f*_P_ of the internal standard remained quite constant, ranging from 2.4 to 3.8%. The variation of *f*_P_ in the experimental samples was presumably not caused by variation of TSPO within white blood cells and platelets, as they compose a different compartment from the plasma. Radioligands, like other drugs, bind to numerous plasma proteins, and this binding rapidly equilibrates with the free concentration in plasma water. Predictably, the administration of the blocking agent also modified the plasma measurements of [^18^F]SF51, which showed higher concentrations compared to baseline and a higher *f*_P_, likely due to the displacement of the radioligand from peripheral binding sites; as an example, alpha-1-acid glycoprotein is one such component [23] for which drug-binding can be displaceable. This suggests that *f*_P_ can significantly increase in the presence of pharmacological dose of blocker [24].

Echoing the results of the present study, a new TSPO tracer, [^18^F]BIBD-239, was recently synthesized by introducing fluorine atoms into the aliphatic side chain of the ER176 terminal group [25]. In vitro competition binding assays showed that [^18^F]BIBD-239 has high affinity to TSPO, and animal models of stroke and glioma showed high and displaceable uptake in the lesions, although no significant displacement was observed in the healthy areas of the rat brain [25]. Although molecular docking calculations suggested that [^18^F]BIBD-239 might be insensitive to genotype, this can only be proven with human studies. It should be noted that ER176, whose structure is similar to that of [^18^F]BIBD-239, was initially thought to be insensitive to genotype on the basis of in vitro binding assays [9] but was subsequently discovered to be sensitive when injected into humans [10]. Similarly, the present study cannot determine whether SF51 is sensitive to genotype in humans, but we expect it to be. Indeed, in a competition binding assay against [^3^H]PK11195 using human brain homogenates, the *K*i ratio of SF51 between LAB and HAB was found to be 2.74, which is higher than that reported for ER176 (1.28) [9, 11]. If our future study in humans finds that [^18^F]SF51 is also sensitive to genotype, we nevertheless hope that—as with [^11^C]ER176—the specific binding of [^18^F]SF51 will be high enough to image LABs.

Finally, it should be noted that while good imaging characteristics have been demonstrated in normal mice and monkeys, further studies in both animal models and humans should assess the ability of [^18^F]SF51 to visualize inflammatory responses in pathological conditions.

## Conclusion

This PET imaging study demonstrated that [^18^F]SF51 is an excellent radioligand that can quantify TSPO with a good ratio of specific to nondisplaceable uptake and has minimal radiometabolite accumulation in brain. Radiolabeling with ^18^F renders this ligand suitable for widespread use. Based on these findings, [^18^F]SF51 appears to be a promising next-generation TSPO PET ligand and warrants further evaluation in humans.

## Supplementary Information

Below is the link to the electronic supplementary material.

Supplementary file1 (DOCX 1379 kb)

## Data Availability

The datasets generated during and/or analyzed during the current study are available from the corresponding author on reasonable request.

## References

[1] Nutma E, Fancy N, Weinert M, Marzin MC, Tsartsalis S, Muirhead RCJ, et al. Translocator protein is a marker of activated microglia in rodent models but not human neurodegenerative diseases. bioRxiv. 2022:2022.05.11.491453. 10.1101/2022.05.11.491453. 10.1038/s41467-023-40937-z PMC10462763 37640701

[2] Kreisl WC, Kim MJ, Coughlin JM, Henter ID, Owen DR, Innis RB (2020). PET imaging of neuroinflammation in neurological disorders. Lancet Neurol.

[3] Shetty HU, Zoghbi SS, Morse CL, Kowalski A, Hirvonen J, Innis RB (2020). Development of a non-radiometric method for measuring the arterial input function of a (11)C-labeled PET radiotracer. Sci Rep.

[4] Charbonneau P, Syrota A, Crouzel C, Valois JM, Prenant C, Crouzel M (1986). Peripheral-type benzodiazepine receptors in the living heart characterized by positron emission tomography. Circulation.

[5] Fujita M, Kobayashi M, Ikawa M, Gunn RN, Rabiner EA, Owen DR (2017). Comparison of four (11)C-labeled PET ligands to quantify translocator protein 18 kDa (TSPO) in human brain: (R)-PK11195, PBR28, DPA-713, and ER176-based on recent publications that measured specific-to-non-displaceable ratios. EJNMMI Res.

[6] Briard E, Zoghbi SS, Imaizumi M, Gourley JP, Shetty HU, Hong J (2008). Synthesis and evaluation in monkey of two sensitive 11C-labeled aryloxyanilide ligands for imaging brain peripheral benzodiazepine receptors in vivo. J Med Chem.

[7] James ML, Fulton RR, Henderson DJ, Eberl S, Meikle SR, Thomson S (2005). Synthesis and in vivo evaluation of a novel peripheral benzodiazepine receptor PET radioligand. Bioorg Med Chem.

[8] Kreisl WC, Fujita M, Fujimura Y, Kimura N, Jenko KJ, Kannan P (2010). Comparison of [(11)C]-(R)-PK 11195 and [(11)C]PBR28, two radioligands for translocator protein (18 kDa) in human and monkey: Implications for positron emission tomographic imaging of this inflammation biomarker. Neuroimage.

[9] Zanotti-Fregonara P, Zhang Y, Jenko KJ, Gladding RL, Zoghbi SS, Fujita M (2014). Synthesis and evaluation of translocator 18 kDa protein (TSPO) positron emission tomography (PET) radioligands with low binding sensitivity to human single nucleotide polymorphism rs6971. ACS Chem Neurosci.

[10] Ikawa M, Lohith TG, Shrestha S, Telu S, Zoghbi SS, Castellano S (2017). 11C-ER176, a Radioligand for 18-kDa Translocator Protein, Has Adequate Sensitivity to Robustly Image All Three Affinity Genotypes in Human Brain. J Nucl Med.

[11] Siméon FG, Lee JH, Morse CL, Stukes I, Zoghbi SS, Manly LS (2021). Synthesis and screening in mice of fluorine-containing PET radioligands for TSPO: discovery of a promising (18)F-labeled ligand. J Med Chem.

[12] Lee JH, Simeon FG, Liow JS, Morse CL, Gladding RL, Montero Santamaria JA (2022). In vivo evaluation of six analogs of (11)C-ER176 as candidate (18)F-labeled radioligands for translocator protein 18 kDa (TSPO). J Nucl Med.

[13] Mansour N, Al-Shamrani S, editors. Science education in the Arab Gulf states: visions, sociocultural contexts and challenges. Rotterdam: SensePublishers; 2015

[14] Percie du Sert N, Hurst V, Ahluwalia A, Alam S, Avey MT, Baker M (2020). The ARRIVE guidelines 2.0: Updated guidelines for reporting animal research. PLoS Biol..

[15] Camsonne R, Crouzel C, Comar D, Maziere M, Prenant C, Sastre J (1984). Synthesis of N-(11C) methyl, N-(methyl-1 propyl),(chloro-2 phenyl)-1 isoquinoleine carboxamide-3 (PK 11195): a new ligand for peripheral benzodiazepine receptors. J Labelled Compd Radiopharm.

[16] Lee JH, Siméon FG, Liow JS, Morse CL, Gladding RL, Santamaria JAM (2022). In Vivo Evaluation of 6 Analogs of (11)C-ER176 as Candidate (18)F-Labeled Radioligands for 18-kDa Translocator Protein. J Nucl Med.

[17] Zoghbi SS, Shetty HU, Ichise M, Fujita M, Imaizumi M, Liow JS (2006). PET imaging of the dopamine transporter with 18F-FECNT: a polar radiometabolite confounds brain radioligand measurements. J Nucl Med.

[18] Gandelman MS, Baldwin RM, Zoghbi SS, Zea-Ponce Y, Innis RB (1994). Evaluation of ultrafiltration for the free-fraction determination of single photon emission computed tomography (SPECT) radiotracers: beta-CIT, IBF, and iomazenil. J Pharm Sci.

[19] Cunningham VJ, Rabiner EA, Slifstein M, Laruelle M, Gunn RN (2010). Measuring drug occupancy in the absence of a reference region: the Lassen plot re-visited. J Cereb Blood Flow Metab.

[20] Lassen NA, Bartenstein PA, Lammertsma AA, Prevett MC, Turton DR, Luthra SK (1995). Benzodiazepine receptor quantification in vivo in humans using [11C]flumazenil and PET: application of the steady-state principle. J Cereb Blood Flow Metab.

[21] Imaizumi M, Briard E, Zoghbi SS, Gourley JP, Hong J, Fujimura Y (2008). Brain and whole-body imaging in nonhuman primates of [11C]PBR28, a promising PET radioligand for peripheral benzodiazepine receptors. Neuroimage.

[22] Pike VW (2009). PET radiotracers: crossing the blood-brain barrier and surviving metabolism. Trends Pharmacol Sci.

[23] Lockhart A, Davis B, Matthews JC, Rahmoune H, Hong G, Gee A (2003). The peripheral benzodiazepine receptor ligand PK11195 binds with high affinity to the acute phase reactant alpha1-acid glycoprotein: implications for the use of the ligand as a CNS inflammatory marker. Nucl Med Biol.

[24] Huang Z, Ung T (2013). Effect of alpha-1-acid glycoprotein binding on pharmacokinetics and pharmacodynamics. Curr Drug Metab.

[25] Chen H, Jiang Z, Cheng X, Zheng W, Sun Y, Yu Z (2022). [(18)F]BIBD-239: (18)F-Labeled ER176, a Positron Emission Tomography Tracer Specific for the Translocator Protein. Mol Pharm.

