# Supplementary material for: In vivo evaluation of a novel 18F-labeled PET radioligand for translocator protein 18 kDa (TSPO) in monkey brain
Source: Eur J Nucl Med Mol Imaging. 2023 May 30;50(10):2962–70. doi: 10.1007/s00259-023-06270-9 (PMC10382351; doi:10.1007/s00259-023-06270-9)
Supplement: Supplementary file 1 — Supplementary file1 (DOCX 1379 kb) [file 259_2023_6270_MOESM1_ESM.docx]

***In vivo* evaluation of a novel ^18^F-labeled PET radioligand for translocator protein 18kDa (TSPO) in monkey brain**

Xuefeng Yan, Fabrice G. Siméon, Jeih-San Liow, Cheryl L. Morse, Jose A. Montero Santamaria, Madeline Jenkins, Lester S. Manly, Maia Van Buskirk, Sami S. Zoghbi,

Victor W. Pike, Robert B. Innis, and Paolo Zanotti-Fregonara

Submitted to *EJNMMI* as an Original Article, October 2022

**Corresponding author:** Xuefeng Yan, MD, PhD, xuefeng.yan@nih.gov

**Supplementary Information**

**Supplementary Table S1.** Summary of experimental protocols for baseline and pre-blocked scans of [^18^F]SF51.

| **Parameter** |  | Monkey 1 | | **Monkey 2** | | | | Monkey 3 | | |
| --- | --- | --- | --- | --- | --- | --- | --- | --- | --- | --- |
|  |  | Baseline | Blocked |  | Baseline | Blocked |  | | Baseline | Blocked |
| Body weight  (kg) |  | 11.0 | 11.0 |  | 9.6 | 9.9 |  | | 10.8 | 10.9 |
| Injected activity (MBq) |  | 168 | 195 |  | 167 | 257 |  | | 199 | 202 |
| Molar activity (µCi/nmol) |  | 2494 | 3423 |  | 3158 | 3328 |  | | 2393 | 2498 |
| Injected mass (nmol/kg) |  | 0.25 | 0.14 |  | 0.15 | 0.14 |  | | 0.21 | 0.2 |

**Supplementary Table S2.** Thirty-three predefined regions of interest were used for data analysis, which were merged into the eleven final regions.

|  | **Pre-defined region** | **Left-right merge** | **Combined region** | **Reported region** |
| --- | --- | --- | --- | --- |
| 1 | Right_Prefrontal Gyrus | Prefrontal Gyrus | Frontal Cortex | Frontal Cortex |
| 2 | Left_Prefrontal Gyrus |  |  |  |
| 3 | Right_Basofrontal Gyrus | Basofrontal Gyrus |  |  |
| 4 | Left_Basofrontal Gyrus |  |  |  |
| 5 | Right_Anterior Cingulate Cortex | Anterior Cingulate Cortex | Cingulate Cortex | Cingulate Cortex |
| 6 | Left_Anterior Cingulate Cortex |  |  |  |
| 7 | Right_Posterior Cingulate Cortex | Posterior Cingulate Cortex |  |  |
| 8 | Left_Posterior Cingulate Cortex |  |  |  |
| 9 | Right_Caudate Nucleus | Caudate Nucleus | Striatum | Striatum |
| 10 | Left_Caudate Nucleus |  |  |  |
| 11 | Right_Putamen | Putamen |  |  |
| 12 | Left_Putamen |  |  |  |
| 13 | Right_Insula | Insula | Insula | Insula |
| 14 | Left_Insula |  |  |  |
| 15 | Right_Lateral Temporal Lobe | Lateral Temporal Lobe | Temporal Cortex | Temporal Cortex |
| 16 | Left_Lateral Temporal Lobe |  |  |  |
| 17 | Right_Medial Temporal Lobe | Medial Temporal Lobe |  |  |
| 18 | Left_Medial Temporal Lobe |  |  |  |
| 19 | Right_Amygdala | Amygdala | Amygdala | Amygdala |
| 20 | Left_Amygdala |  |  |  |
| 21 | Right_Hippocampus | Hippocampus | Hippocampus | Hippocampus |
| 22 | Left_Hippocampus |  |  |  |
| 23 | Right_Thalamus | Thalamus | Thalamus | Thalamus |
| 24 | Left_Thalamus |  |  |  |
| 25 | Right_Hypothalamus | Hypothalamus | Hypothalamus |  |
| 26 | Left_Hypothalamus |  |  |  |
| 27 | Right_Parietal Lobe | Parietal Lobe | Parietal Cortex | Parietal Cortex |
| 28 | Left_Parietal Lobe |  |  |  |
| 29 | Right_Occipital Lobe | Occipital Lobe | Occipital Cortex | Occipital Cortex |
| 30 | Left_Occipital Lobe |  |  |  |
| 31 | Cerebellum | Cerebellum | Cerebellum | Cerebellum |
| 32 | Pons | Pons | Pons |  |
| 33 | Midbrain | Midbrain | Midbrain |  |

**Supplementary Table S3.** Regional distribution volume corrected for plasma protein binding (*V*_T_/*f*_P_) of [^18^F]SF51 in monkey brain (n=3) using Logan plot.

| Region |  | *V*_T_/*f*_P_ (mL·cm^-3^) | | | | |  | *t** (min) | | | | |  | *(Logan-2TCM)/2TCM × 100 %* | | | | |
| --- | --- | --- | --- | --- | --- | --- | --- | --- | --- | --- | --- | --- | --- | --- | --- | --- | --- | --- |
|  |  | Baseline | |  | Blocked | |  | Baseline | |  | Blocked | |  | Baseline | |  | Blocked | |
|  |  | Mean | SD |  | Mean | SD |  | Mean | SD |  | Mean | SD |  | Mean | SD |  | Mean | SD |
| Whole Brain |  | 194 | 15 |  | 24 | 8 |  | 30 | 0 |  | 17 | 12 |  | -4.4 | 0.7 |  | -1.8 | 2.6 |
| Frontal Cortex |  | 192 | 12 |  | 25 | 8 |  | 30 | 0 |  | 22 | 8 |  | -4.3 | 0.6 |  | -2.6 | 2.3 |
| Cingulate Cortex |  | 208 | 16 |  | 24 | 8 |  | 28 | 3 |  | 23 | 8 |  | -4.6 | 0.6 |  | -2.6 | 2.6 |
| Striatum |  | 215 | 16 |  | 25 | 8 |  | 28 | 3 |  | 16 | 9 |  | -4.4 | 0.8 |  | -1.5 | 3.2 |
| Insula |  | 214 | 14 |  | 23 | 8 |  | 28 | 3 |  | 12 | 7 |  | -4.3 | 0.2 |  | -2.4 | 2.0 |
| Temporal Cortex |  | 201 | 21 |  | 22 | 8 |  | 33 | 6 |  | 19 | 11 |  | -4.2 | 0.7 |  | -1.7 | 2.2 |
| Amygdala |  | 229 | 20 |  | 23 | 6 |  | 30 | 0 |  | 19 | 11 |  | -5.5 | 0.6 |  | -0.3 | 4.0 |
| Hippocampus |  | 211 | 24 |  | 21 | 7 |  | 37 | 6 |  | 14 | 10 |  | -4.1 | 0.7 |  | -1.3 | 2.8 |
| Thalamus |  | 208 | 20 |  | 26 | 8 |  | 28 | 10 |  | 15 | 11 |  | -4.4 | 0.6 |  | -4.9 | 3.2 |
| Parietal Cortex |  | 193 | 11 |  | 23 | 7 |  | 27 | 3 |  | 16 | 14 |  | -4.4 | 0.6 |  | -2.1 | 2.2 |
| Occipital Cortex |  | 175 | 14 |  | 22 | 7 |  | 33 | 6 |  | 18 | 10 |  | -4.4 | 0.6 |  | -1.7 | 2.3 |
| Cerebellum |  | 172 | 19 |  | 25 | 8 |  | 43 | 6 |  | 14 | 6 |  | -4.2 | 1.0 |  | -2.6 | 2.4 |

SD: standard deviation.

**Supplementary Table S4.** Estimated *K*_1_ values of the various regions (mL·cm^-3^·min^-1^) from baseline and blocked scans of three rhesus monkeys using 2TCM.

| Regions |  | Baseline | | |  | Blocked | | |
| --- | --- | --- | --- | --- | --- | --- | --- | --- |
|  |  | Mean | SD | COV |  | Mean | SD | COV |
| Whole Brain |  | 0.31 | 0.03 | 0.11 |  | 0.41 | 0.07 | 0.17 |
| Frontal Cortex |  | 0.30 | 0.04 | 0.14 |  | 0.36 | 0.06 | 0.18 |
| Cingulate Cortex |  | 0.30 | 0.06 | 0.18 |  | 0.34 | 0.07 | 0.20 |
| Striatum |  | 0.38 | 0.06 | 0.16 |  | 0.65 | 0.04 | 0.07 |
| Insula |  | 0.35 | 0.05 | 0.13 |  | 0.37 | 0.02 | 0.05 |
| Temporal Cortex |  | 0.30 | 0.04 | 0.13 |  | 0.36 | 0.07 | 0.21 |
| Amygdala |  | 0.29 | 0.03 | 0.10 |  | 0.38 | 0.03 | 0.08 |
| Hippocampus |  | 0.33 | 0.03 | 0.09 |  | 0.48 | 0.06 | 0.13 |
| Thalamus |  | 0.35 | 0.05 | 0.16 |  | 0.50 | 0.08 | 0.16 |
| Parietal Cortex |  | 0.30 | 0.04 | 0.14 |  | 0.35 | 0.06 | 0.17 |
| Occipital Cortex |  | 0.31 | 0.03 | 0.10 |  | 0.40 | 0.04 | 0.10 |
| Cerebellum |  | 0.34 | 0.07 | 0.20 |  | 0.62 | 0.10 | 0.16 |

COV: coefficient of variation; SD: standard deviation.

**Supplementary Table S5.** Regional brain distribution volume corrected for plasma protein binding (*V*_T_/*f*_P_) of [^18^F]SF51 after PBR28 pre-blockade (5 mg/kg) in one monkey. *f*_Pbase_: 5.7%; *f*_Pblock_: 19.3%

| Region |  | *V*_T_/*f*_P_ (mL·cm^-3^) | | |
| --- | --- | --- | --- | --- |
|  |  | Baseline |  | Blocked |
| Whole Brain |  | 240 |  | 10 |
| Frontal Cortex |  | 241 |  | 10 |
| Cingulate Cortex |  | 260 |  | 10 |
| Striatum |  | 266 |  | 10 |
| Insula |  | 264 |  | 10 |
| Temporal Cortex |  | 241 |  | 9 |
| Amygdala |  | 284 |  | 9 |
| Hippocampus |  | 253 |  | 9 |
| Thalamus |  | 259 |  | 12 |
| Parietal Cortex |  | 246 |  | 10 |
| Occipital Cortex |  | 217 |  | 10 |
| Cerebellum |  | 208 |  | 10 |

**
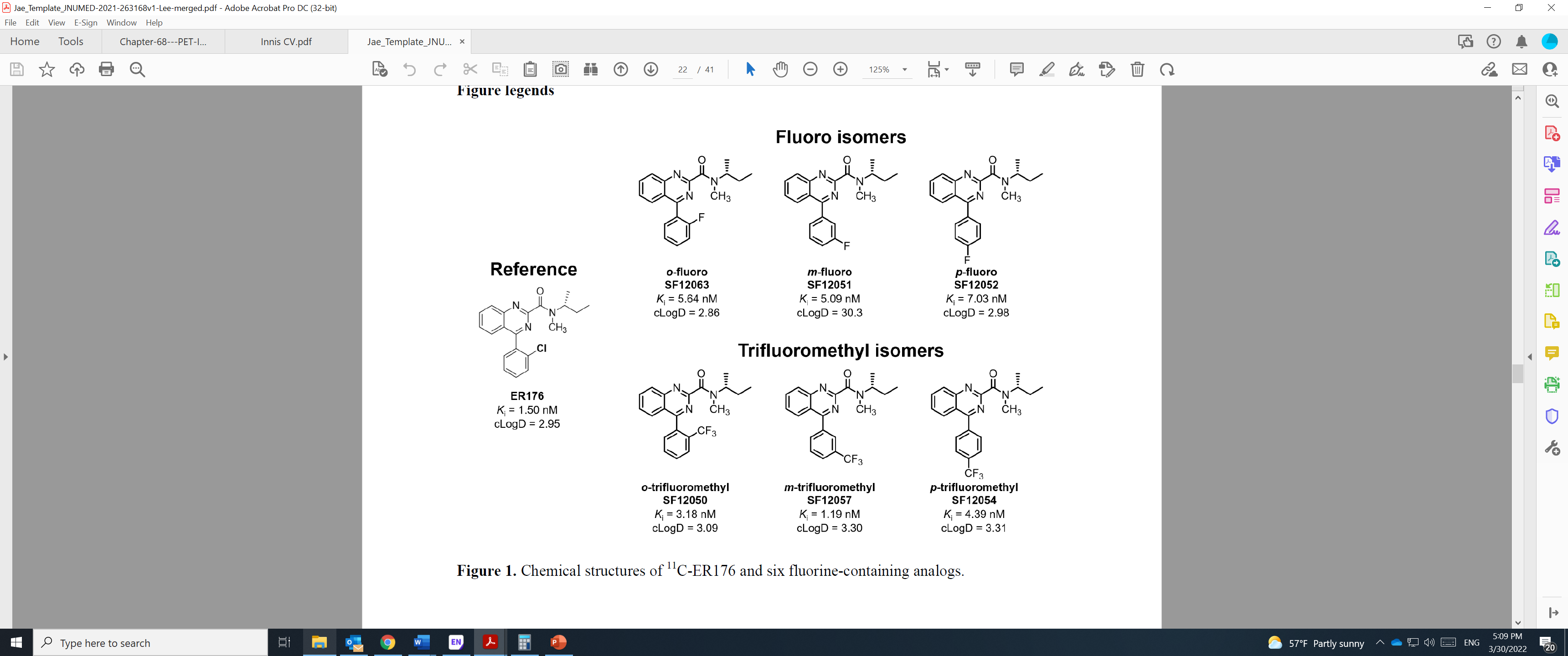
**

**Supplementary Figure S1**. Chemical structures of [^11^C]ER176 and six fluorine-containing analogs. This figure was originally published in: Lee JH, Siméon FG, Liow JS, Morse CL, Gladding RL, Santamaria JAM, Henter ID, Zoghbi SS, Pike VW, Innis RB. In Vivo Evaluation of 6 Analogs of [^11^C]ER176 as Candidate ^18^F-Labeled Radioligands for 18-kDa Translocator Protein. J Nucl Med. 2022 Aug;63(8):1252-1258. © SNMMI


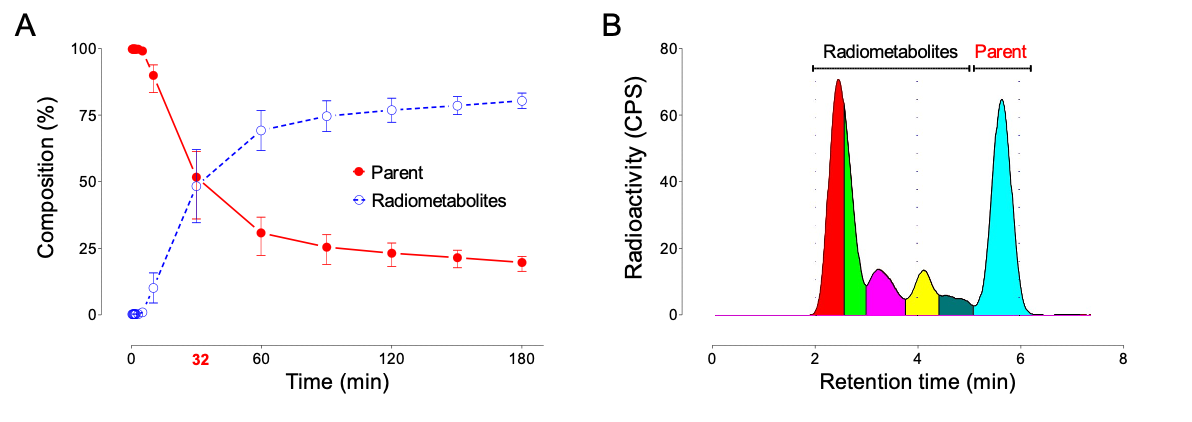


**Supplementary Figure S2.** **(A)** The percentage of blood components for baseline scans changed over time. Point and error bars, respectively, represent the mean and range of three scans. **(B)** Representative radiochromatogram showing the arterial plasma composition profile after i.v. injection of [^18^F]SF51 into a monkey. During the baseline study, at 30 minutes the parent was 36% while the total radiometabolites were 64%. There were more than five radiometabolites in plasma, although they were not well separated. The parent was well separated from the radiometabolites.


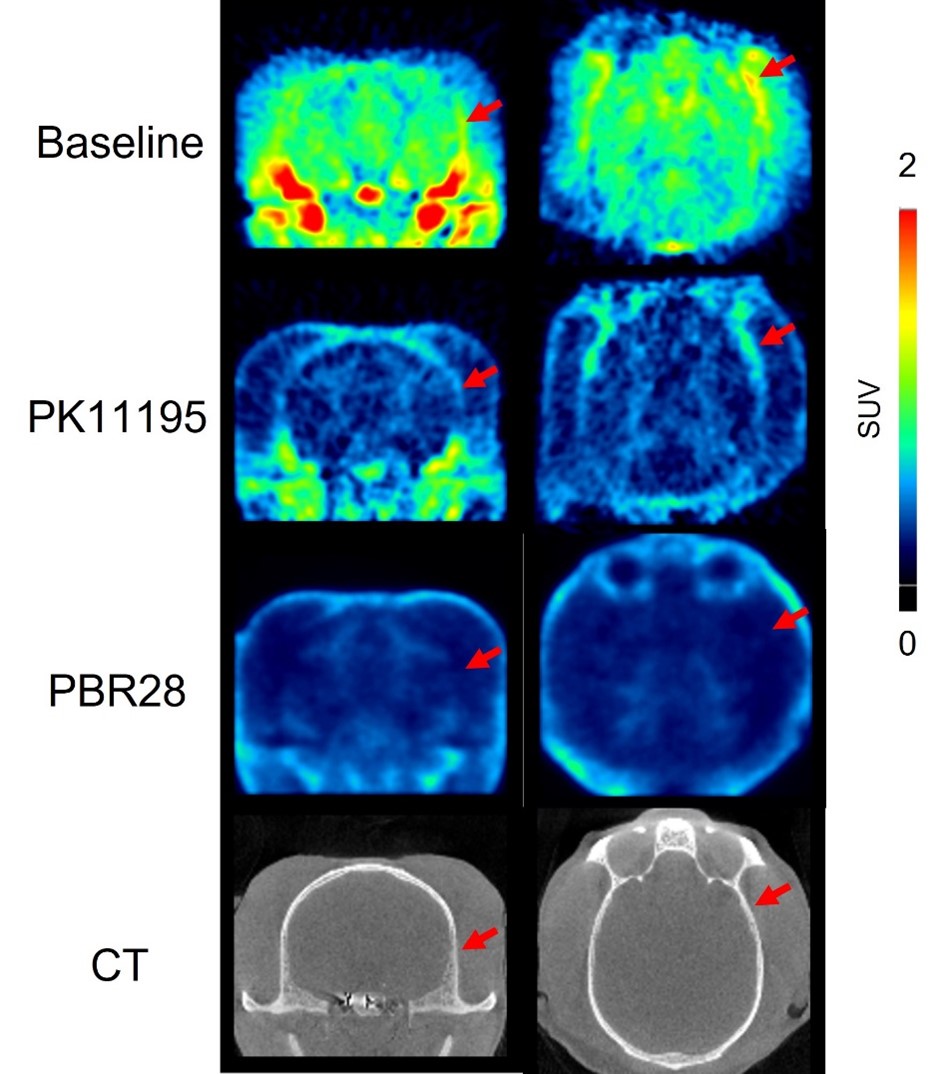


**Supplementary Figure S3.** The SUV PET images were generated from a 90-120-minute scan. [^18^F]SF51 in monkey brain at baseline (top row), after PK11195 (5 mg/kg, middle top row) and PBR28 blockade (5 mg/kg, middle bottom row). The individual monkey’s CT is shown in the bottom row. Skull is indicated with red arrows.


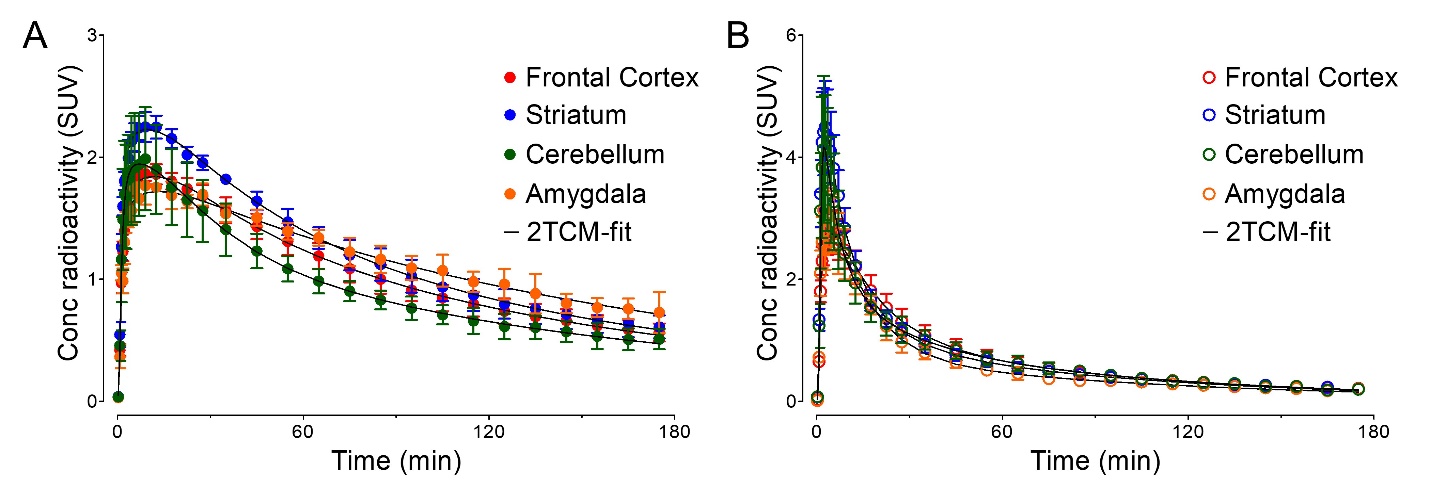


**Supplementary Figure S4.** Concentration of radioactivity in rhesus macaques at baseline **(A)** and after pre-blockade with PK11195 **(B)**. The selective 18kDa translocator protein (TSPO) ligand PK11195 (5 mg/kg i.v.) was injected 10 minutes prior to [^18^F]SF51. Concentration was expressed as standardized uptake value (SUV). Symbols and error bars represent mean and SD (n=3), respectively.

**
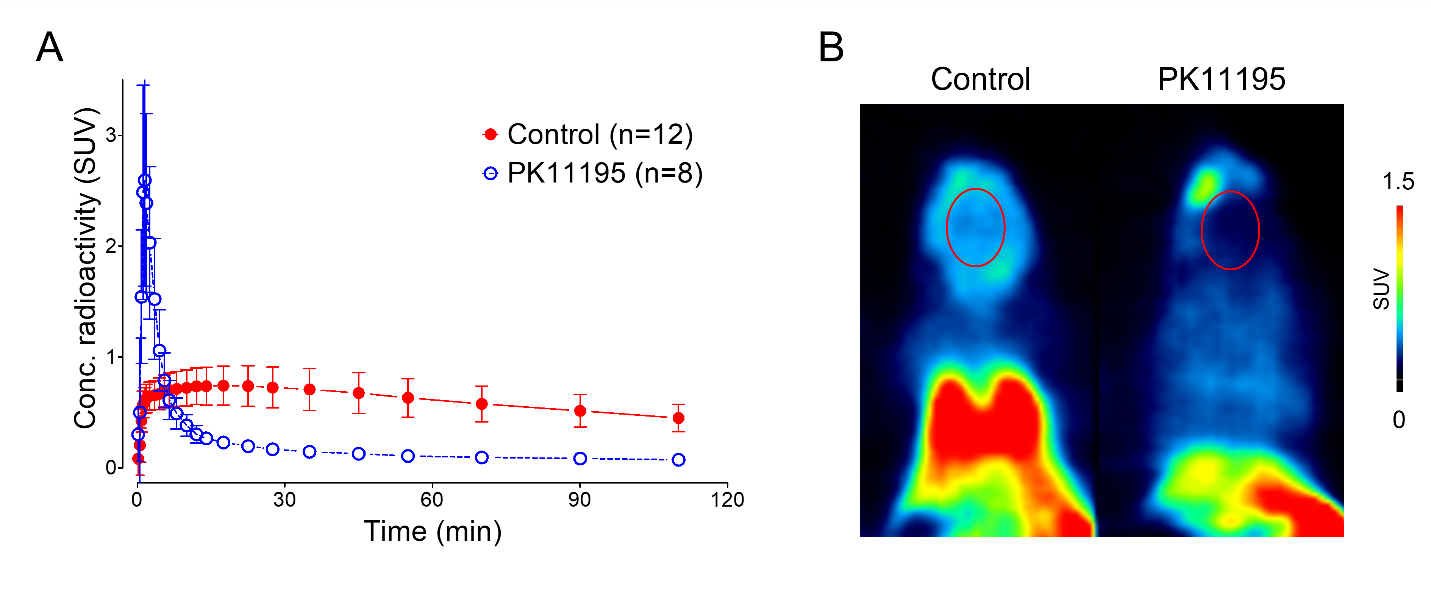
**

**Supplementary Figure S5**. Time-activity curves of whole-brain uptake **(A)** and representative standardized uptake value (SUV) images **(B)** in mice. PK11195 (5 mg/kg i.v.) was injected 10 minutes prior to [^18^F]SF51. SUV images were averaged over the last 40 minutes (60-100 minutes) of scan duration. The brain region is circled in red.


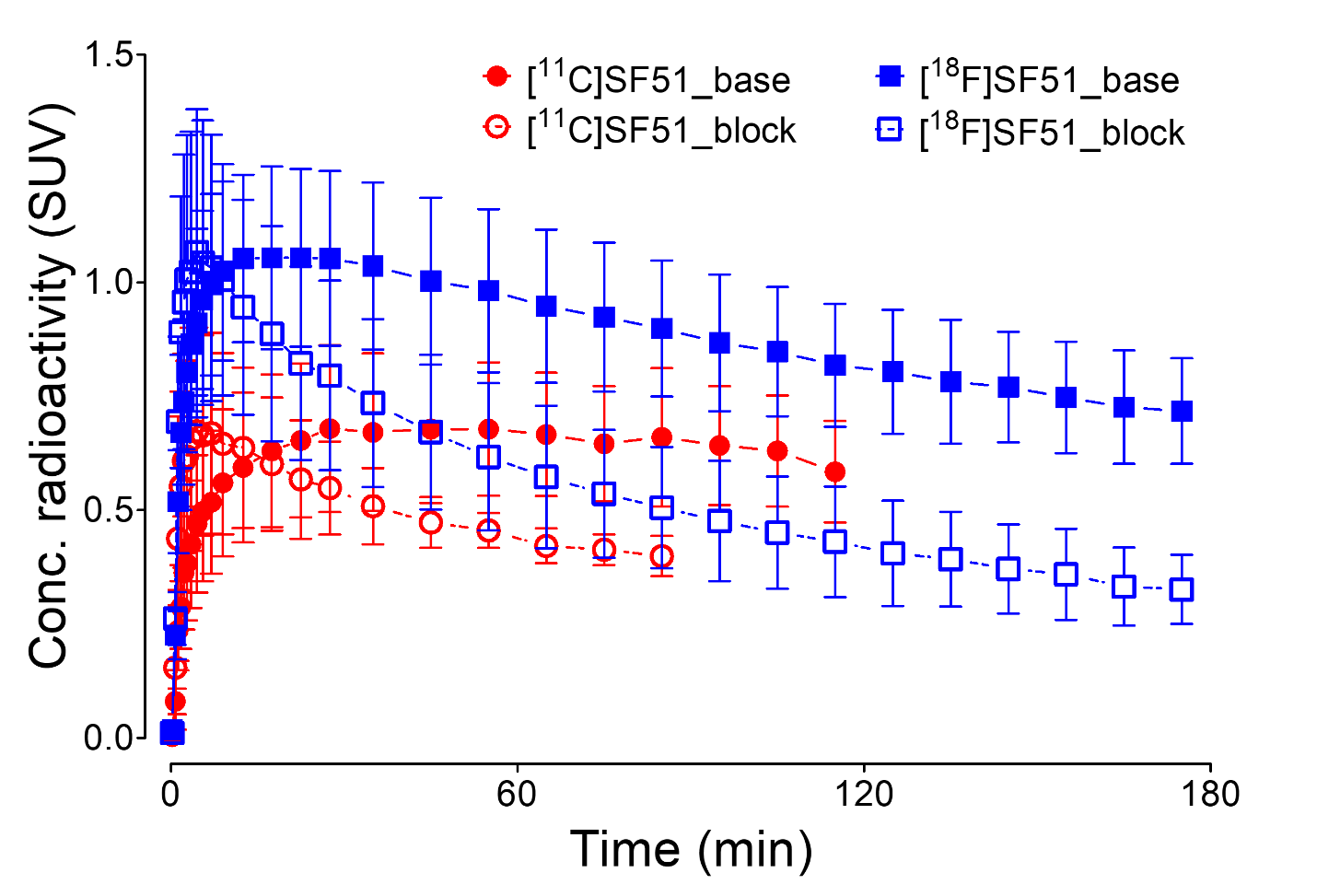


**Supplementary Figure S6**. Time-activity curves of skull uptake at baseline and after pre-blockade with PK11195. The selective 18kDa translocator protein (TSPO) ligand PK11195 (5 mg/kg i.v.) was injected 10 minutes prior to radioligand, [^11^C]SF51 or [^18^F]SF51. Concentration was expressed as standardized uptake value (SUV). Symbols and error bars represent mean and SD (n=3), respectively. The blockable uptake in the skull was visible with both [^11^C]SF51 and [^18^F]SF51.
